# Effects of Knots on Protein Folding Properties

**DOI:** 10.1371/journal.pone.0074755

**Published:** 2013-09-04

**Authors:** Miguel A. Soler, Patrícia F. N. Faísca

**Affiliations:** 1 Centro de Física da Matéria Condensada, Universidade de Lisboa, Lisboa, Portugal; 2 Departamento de Física, Universidade de Lisboa, Lisboa, Portugal; University of Leeds, United Kingdom

## Abstract

This work explores the impact of knots, knot depth and motif of the threading terminus in protein folding properties (kinetics, thermodynamics and mechanism) via extensive Monte Carlo simulations of lattice models. A knotted backbone has no effect on protein thermodynamic stability but it may affect key aspects of folding kinetics. In this regard, we found clear evidence for a functional advantage of knots: knots enhance kinetic stability because a knotted protein unfolds at a distinctively slower rate than its unknotted counterpart. However, an increase in knot deepness does not necessarily lead to more effective changes in folding properties. In this regard, a terminus with a non-trivial conformation (e.g. hairpin) can have a more dramatic effect in enhancing kinetic stability than knot depth. Nevertheless, our results suggest that the probability of the denatured ensemble to keep knotted is higher for proteins with deeper knots, indicating that knot depth plays a role in determining the topology of the denatured state. Refolding simulations starting from denatured knotted conformations show that not every knot is able to nucleate folding and further indicate that the formation of the knotting loop is a key event in the folding of knotted trefoils. They also show that there are specific native contacts within the knotted core that are crucial to keep a native knotting loop in denatured conformations which otherwise have no detectable structure. The study of the knotting mechanism reveals that the threading of the knotting loop generally occurs towards late folding in conformations that exhibit a significant degree of structural consolidation.

## Introduction

Proteins whose backbone is tangled in a topological knot represent extreme cases of geometric complexity. A recent study by Sulkowska *et al*. [1] concluded that knotted proteins and proteins with slipknots [2] in their native structures represent about 1% of the protein data bank (PDB). The most complex knotted protein found to date in this subset of the PDB is the Stevedore's (or 6_1_) knot [3] while the most ubiquitous knotted motif is the simpler trefoil (or 3_1_) knot [4]–[6]. Since their discovery knotted proteins have attracted considerable attention. In particular, researchers have directed their efforts towards solving two major questions: 1) how do knotted proteins fold? [7], [8] and 2) what kind of functional advantage is conferred to proteins that have a knot in their native structure? [9]

With a few exceptions [3], [10], most research efforts have focused on the investigation of proteins with trefoil knots. Seminal studies by Jackson and collaborators, which explored proteins YibK and YbeA [11], [12], highlighted the formation of folding intermediates and parallel folding pathways in a rather complex self-assembly process, where knotting appeared to take place in a denatured-like state followed by the structural consolidation of the knotted region towards late folding [13], [14]. Based on the observation that knotted loops could persist in unstructured chemically denatured states Jackson *et al*. later proposed that the in vitro folding efficiency of YibK and YbeA could be a direct consequence of the high prevalence of denatured-state knots [15]. Recently, an illuminating experiment by the same laboratory, which used a cell-free translation system, showed that newly translated polypeptide chains (which are necessarily unknotted) can actually tangle spontaneously and without forming misfolded states, thus challenging the view that loose knots existing in the denatured ensembles of YibK and YbeA are pre-requisite to folding success of these particular knotted proteins [16]. However, and in line with other experimental records [17], these experiments also showed very clearly that the folding speed of unfolded (and, most importantly, unknotted) chains is very slow (and up to 1.5 orders of magnitude slower than that registered in refolding experiments following chemical denaturation). Furthermore, and most interestingly, the knotting process is significantly accelerated by the GroEL-GroEs chaperonin system, suggesting they may play a major role in vivo for this class of bacterial knotted protein.

The detailed microscopic picture gathered from a plethora of simulation studies raging from lattice models [18], [19], to coarse-grained [20]–[22] and atomistic [23], [24] off-lattice models based on native-centric [18], [19], [21], [23], modified native-centric [20], and physics-based force fields [24] highlights the fact that the formation of the knotted trefoil is a rather choreographed process involving the threading of either the protein C-terminus [18], [19], [23], [24] or a slipknot conformation [21], [23], [25] through a knotting loop. For the vast majority of studied proteins knotting and folding were found to be uncoupled processes [18]–[21], [23], [24] but a recent study showed that for the designed protein 2ouf [17] knotting occurs concomitantly with the formation of the transition state ensemble [22].

The picture of the folding mechanism of knotted trefoil proteins outlined above is certainly not complete but is quite promising. Unfortunately, a similar level of advancement was not yet attained regarding the understanding of the functional role played by knots and slipknots in proteins. Based on the analysis of specific knotted systems it has been suggested that knots (and slipknots) could play a role against degradation by the proteasome [26], provide structural stability in transporter proteins [1], enhance thermal [2], [27] and mechanical [28], [29] stability or even alter enzymatic activity [29]. However, as been recently pointed out, in the majority of cases it is not possible to determine the structural and functional advantages provided by knotted folds [1].

In the present work we perform an extensive study based on lattice models and Monte Carlo Simulations to systematically investigate the impact of knot depth on protein folding properties and on the folding mechanism. Despite their simplicity lattice models have been successfully used to explore fundamental aspects of protein folding [30]–[37] and related phenomena [38]–[41]. In particular, their computational feasibility allows for rather accurate estimates of protein thermodynamics and kinetics.

Our starting point in this study is a lattice model protein that contains a shallow trefoil knot and which we investigated in previous reports [18], [19]. This model protein folds efficiently and the knotting mechanics is based on a threading movement of the protein terminus that lays closer to the knotted core. We find that placing the knotted core deeper inside the polypeptide chain by increasing the size of the termini, results into a less efficient folding process, which becomes more prone to topological traps, and with low folding rates. On the other hand, the considerably large reduction we observe for the rates of unfolding indicate that knot deepness is as an efficient source of kinetic stability. Moreover, we found that the knotted backbone is extraordinarily resilient to unknot, which confers a high topological stability to the denatured state. Interestingly, and in line with previous simulation results [23], the occurrence of a non-specific knot in the protein backbone is not enough to nucleate folding. In mechanistic terms we have found evidence for a preferred knotting mechanism based on a threading movement of the chain terminus that is closer to the knotted core through a native-like knotting loop occurring in preordered conformations.

## Model and Methods

### The simple lattice Gō model and Monte Carlo folding simulation

We consider a simple cubic lattice model of a protein molecule with chain length *N*. In the simple lattice representation the protein is reduced to its backbone structure: amino acids, modeled by beads of uniform size, are placed on the vertices of a regular three-dimensional lattice and the peptide bond, which covalently links amino acids along the polypeptide chain, is represented by sticks with uniform (unit) length corresponding to the lattice spacing. The latter represents the distance between two 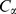 atoms along the polypeptide chain. In order to satisfy excluded volume constraint only one bead is allowed per lattice site. To model protein energetics we use the Gō potential [42], according to which the energy of a conformation, defined by the set of bead coordinates 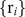, is given by the contact Hamiltonian
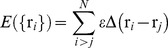
(1)where *ε* is the (uniform) interaction energy parameter (generally taken as −1) and the contact function 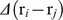, is unity only if beads *i* and *j* form a native contact and is zero otherwise.

In order to mimic the protein's relaxation towards the native state we use the Metropolis Monte Carlo (MC) algorithm [26] together with a local move set that includes corner-flips and end-moves, which displace one bead at a time (the end-moves are exclusively used to move the chain's termini and the corner-flip is used to displace all the other beads in the chain), and the crankshaft move (which involves the simultaneous displacement of two beads except termini beads). The adequacy of the adopted move set to study polymer dynamics including the special case of knotted polymers was established in Ref. [43]. A MC simulation starts from a randomly generated unfolded conformation and folding progress is monitored through several properties (e.g. the fraction of the established native contacts, *Q*). Further details on the adopted simulation algorithm can be found elsewhere [44].

### Folding thermodynamics

In order to explore the thermodynamics of the folding transition and compute equilibrium properties we have conducted very long (10^9^ MC-steps per residue) replica-exchange (RE) MC simulations at 40 different temperatures. Each MC trajectory consists of - at least −10^6^ MC-steps per residue after equilibration. The temperature grid for the RE has been established to ensure a 100% acceptance ratio for the RE-move attempts. To ensure that the replicas equilibrate, we chose a time interval between two consecutive RE-moves which is larger than the largest auto-correlation time for the energy. In the course of a single simulation the replica reliably and repeatedly visits all the temperatures in the grid with cycle time of 40 replica exchange moves, while a single total simulation comprised at least 25 full cycles. This indicates good convergence quality of our simulation data. The results reported here correspond to an average of three RE simulations. The heat capacity 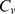 is evaluated from the mean squared fluctuations in energy at each temperature considered in the RE simulations according to the definition
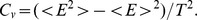



Except otherwise stated all the results presented in this work were evaluated at the melting temperature, 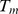. The latter is determined by the co-existence of the lowest energy structure - the native structure - and a multitude of high-energy ones. It is generally estimated from differential scanning calorimetry experiments as being the temperature at which the heat capacity attains its maximum value. For a strict two-state transition (i.e. a folding transition which does not populate intermediate states) 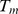 can thus be defined as the temperature at which denatured and native states are equally populated at equilibrium. Throughout the paper the temperature is measured in units of 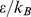, where 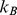 is the Boltzmann constant.

### Kinetics of folding and unfolding

To obtain kinetic properties such as the folding rate, 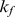, and unfolding rate, 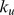, we carried out fixed temperature MC simulations. Folding simulations start from an unfolded conformation and stop when the fraction of formed native contacts *Q* attains one. On the other hand, the unfolding simulations start from the native conformation and stop when the chain attains less than four native contacts. To get statistically significant kinetic measurements, we recorded 2000 independent MC folding runs and 2000 independent MC unfolding runs. The corresponding folding (unfolding) times (i.e. first passage time for folding or unfolding) allow evaluating the distribution of proteins that remain unfolded (folded) as a function of MC ‘time’ (i.e. MC steps). The folding (unfolding) rate is given by the slope of the linear fitting of this distribution to a single-exponential decay.

### Knot detection

The native structure of the knotted protein studied in this work contains a trefoil knot. In order to determine if, whether or not, a conformation sampled in the course of the folding simulation is knotted we used the Koniaris-Muthakumar-Taylor (KMT) algorithm [45], which reduces the lattice backbone to the smallest segment that contains the knot. Details on the adopted procedure can be found elsewhere [18].

### Structural clustering

In order to determine the relevant conformational classes present in an ensemble of conformations we have used the hierarchical clustering algorithm *jclust* available in the MMTSB tool set [46]. Since we are using a lattice model, clustering is done based on contact map similarity. From each identified cluster we extract the conformation that is the closest to the cluster's centroid.

### Statistical error

The statistical error in the measurements reported in the Results section is not shown because it is of the same size or smaller than that of the adopted symbols.

## Results

### Model systems

**Figure 1:**
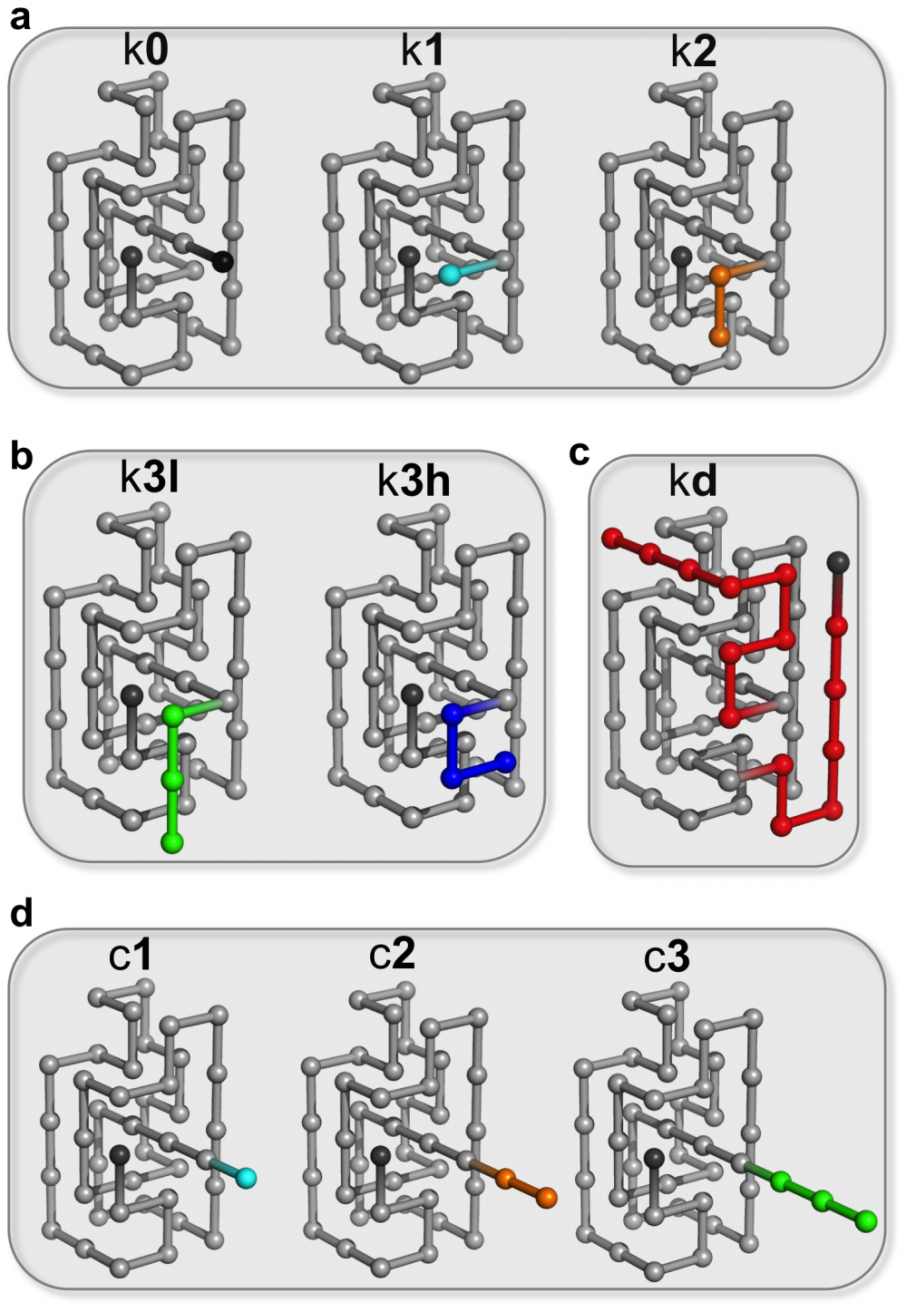
Lattice proteins used in this study that have a trefoil knot. Lattice protein k0 is the reference system that was used to construct the other lattice proteins. The first bead is colored in black and the last bead in grey. The knotted core is located between bead three and bead 22 in k0, which classifies as a shallow knot. In systems k1 and k2 (a) we added one and two beads, respectively, to the first bead to extend the size of the first terminus thus increasing knot depth. The addition of three beads was done in two manners (b). In k3l they form a linear segment and in k3h they are arranged into a hairpin like conformation. In protein kd (c) the size of the first terminus was extended with seven beads and that of the last terminus with eight beads. We have also considered three knotted trefoils where the extended terminus does not establish any native contacts (d). These are used as control systems. Throughout this work we use the color code adopted in these figures to identify each considered lattice system.

In previous studies [18], [19] we explored in detail the folding mechanism of a model protein whose backbone is tangled in a trefoil knot. The minimal segment of the protein backbone that contains the knot (the so-called knotted core) is located between residues three and 22 making it enough to remove three beads in order to untangle the protein. The protein is thus considered to be a shallow knot [9]. This model system (termed k0) together with its unknotted counterpart (u0) (which has the same overall core structure) will be used as template structures in the present work. In particular, in order to evaluate the importance of knot deepness in protein folding we perform a systematic comparison of the folding process of additional pairs of knotted/unknotted native structures that were constructed from the k0/u0 pair by adding additional beads to one or both termini. Specifically, we consider the native conformations k1, k2 and k3 (and their unknotted pairs, u1, u2, and u3) that differ from the corresponding template structures k0 (and u0) by having 1, 2, and 3 extra beads, respectively, added to the terminus that is closest to the knotted core (Figure 1). In the case of k3 we considered two specific conformations: one in which the added beads form a linear segment (k3l) and another one in which they are arranged in a hairpin-like conformation (k3h) (Figure 1B). We also consider an additional knotted backbone where eight beads were added to the first terminus and seven beads to the last terminus (Figure 1C). In this case, in order to form the knot, it is necessary to thread a segment containing 11 beads through a knotting loop. Since this is the deepest knot considered in our work we term it kd. In line with the adopted nomenclature, its unknotted counterpart is termed ud. The threading segment represents 20% of the lattice protein chain's length. Interestingly, in protein YibK (PDB code 1j85), which is considered to be a deep knot, the threading segment (formed by 40 residues) represents 25% of protein's chain length.

The considered systems differ in the location of the knotted core and also in the number of native contacts (1, 2, 3, 4 and 21 for k1, k2, k3l, k3h and kd, respectively). Since the effective formation of these additional native contacts requires the threading of the chain terminus through a knotting loop, their untimely formation may create topological bottlenecks that must be solved by means of backtracking (i.e. the breaking and re-establishment of specific native contacts [21], [47]). In order to investigate how topological bottlenecks and backtracking impart on the folding efficiency of knotted proteins we have compared their folding performance with that of four control systems, c1, c2, c3 and cd, in which the added beads do not establish native interactions with any part of the protein and therefore will not create topological bottlenecks (Figure 1D). The control systems allow assessing the impact of termini length in knotting efficiency.

### Knot deepness and thermal stability

**Figure 2:**
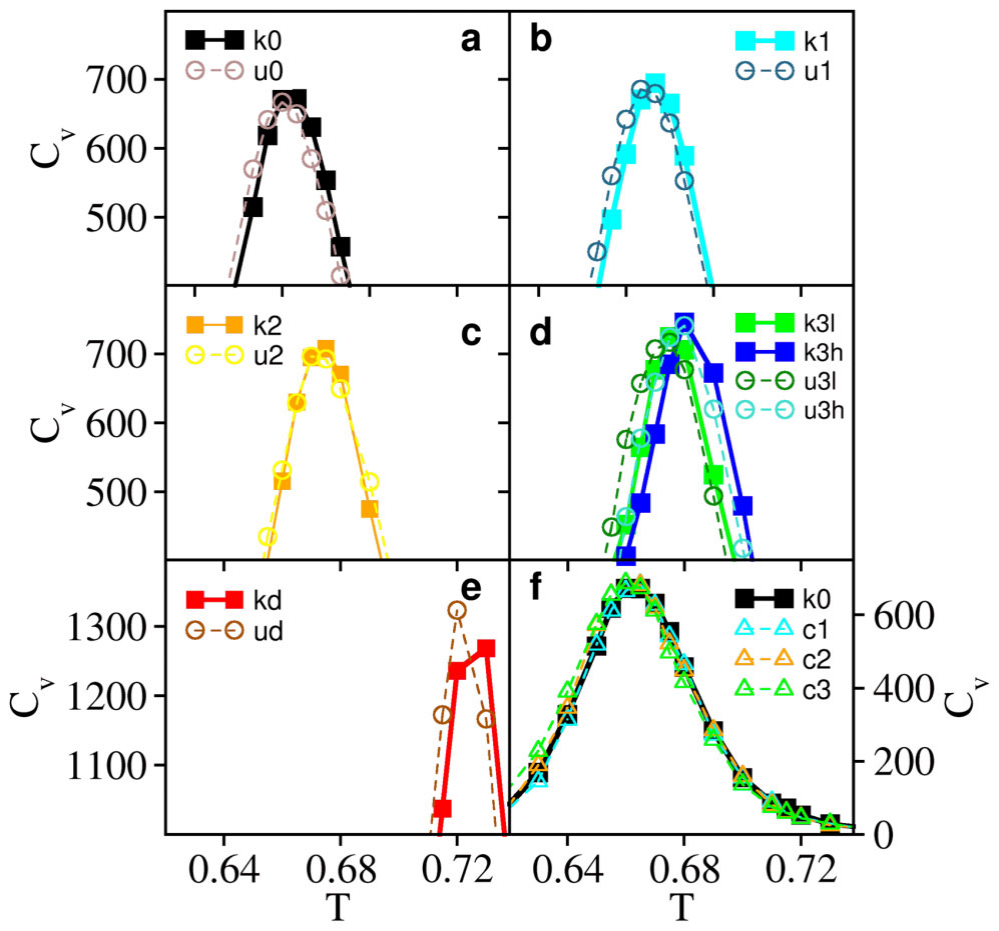
The relation between knot depth and thermodynamic stability. Thermodynamic stability is quantified by the value of the melting temperature *T_m_* (which is the temperature at which the heat capacity peaks). Panels (a) to (e) show a comparison between the peaks of the heat capacity curves of the knotted systems and their unknotted counterparts. Panel (f) reports a direct comparison between the heat capacity curve of the control systems and the reference system k0. Throughout this work squares are used to identify the knotted proteins and circles are used to identify the unknotted ones. The control systems are identified with triangles.

We start by investigating how knot deepness affects thermal stability as quantified by 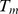. Since the knotted systems have different native state's energies *E* one should not directly compare their thermal stabilities. Instead, one should compare the thermal stability of each knotted structure with that of its unknotted counterpart and determine if an increase in knot deepness leads to a larger difference in *T_m_*. Figure 2A–E reports the location of the heat capacity peak for the six knotted-unknotted native structures considered here. While the 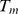 values of the knotted proteins are always slightly larger than those of the unknotted ones the recorded difference is marginal even for the kd-ud pair. In the case of the control systems, i.e., those in which the added beads do not establish additional native contacts a direct comparison of the heat capacity curves is indeed possible and reveals very similar behavior across the considered native structures (Figure 2F). Taken together these results indicate that an increase in knot deepness *per se* does not necessarily lead to an increase in thermal stability.

### Knot deepness and folding rate

**Figure 3:**
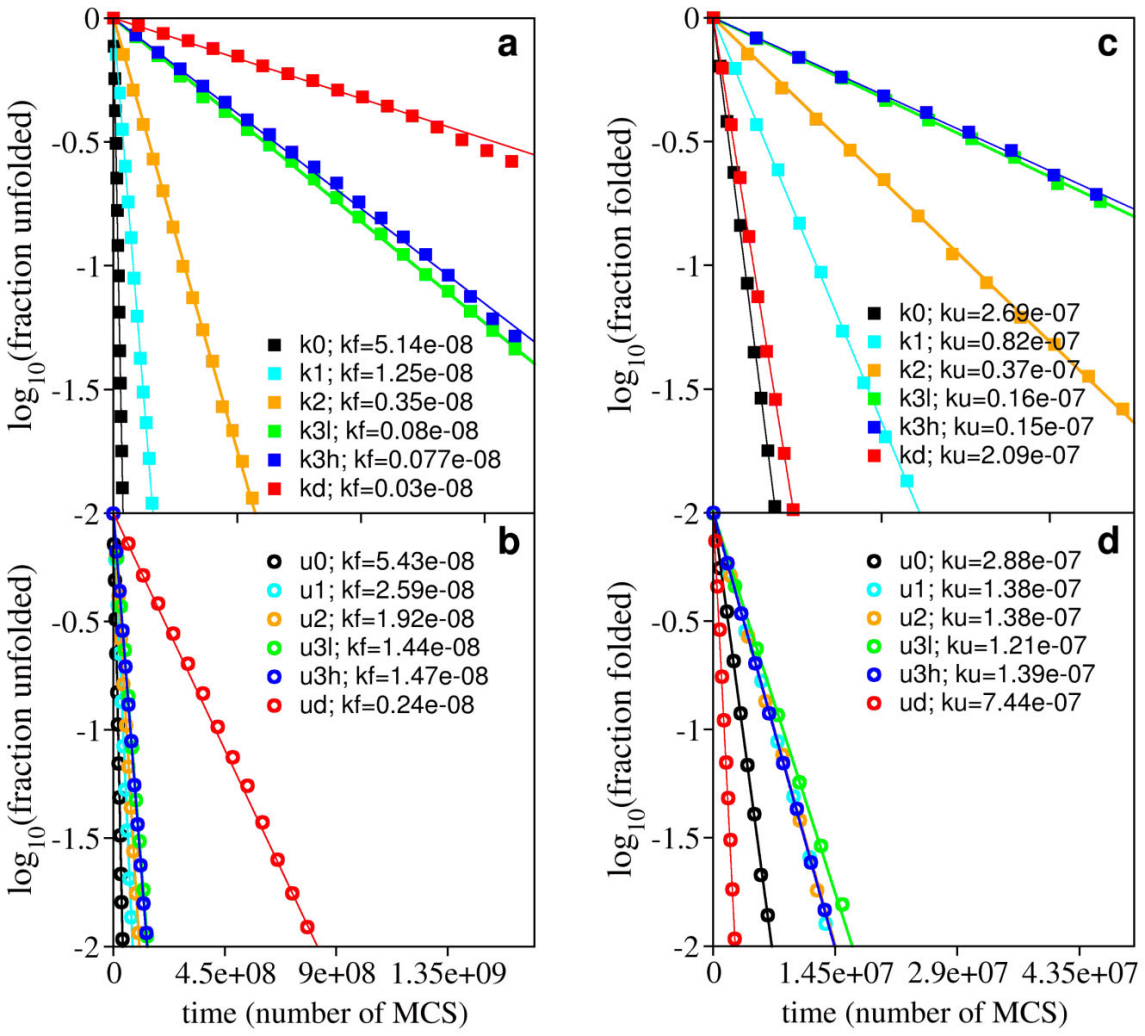
Relation between knot depth, folding rate and unfolding rate. The folding (unfolding) rate corresponds to the slope of each represented curve. The folding rate is determined at 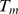 and the unfolding rate slightly above 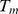. Panel (a) reports the folding rate of the knotted systems and panel (b) reports the folding rate of their unknotted counterparts. Panel (c) reports the unfolding rate of knotted systems, while panel (c) reports the unfolding rate of their unknotted counterparts. The ratio between the folding rate of the deep knot kd and its unfolded counterpart ud is 0.13, and that between the knot k3h and u3h is only 0.05.

The second property we investigate in relation with knot deepness is folding rate. Once again a direct comparison of the folding rate among the knotted systems is not feasible because they exhibit different chain lengths and chain length is known to affect the folding rate of lattice protein systems [44], [48], [49]. Thus, we follow the strategy adopted in the previous section and compare the folding rate of each knotted protein with that of its unknotted counterpart. Our results show that as knot deepness increases the folding rates of the knotted proteins (Figure 3A) become notably smaller than those of their unknotted homologues (Figure 3B). Interestingly, the largest recorded difference is not associated with knot kd (which folds 7.5 times slower than ku) but instead with the knots k3l and k3h, which fold 1.2 and 1.3 orders of magnitude slower than their respective unknots u3l and u3h. This result is somewhat surprising and indicates that the folding mechanism of the k3 knots, especially that of knot k3h, is more prone to error than that of the deepest knot considered here. Thus, the requirement to thread a larger terminus does not necessarily increase the likelihood of topological traps in the folding of knotted proteins. Moreover, since the difference between the k3l and k3h knot is the existence of a structured hairpin-like terminus in k3h we speculate that the latter should play a role in the folding mechanism of this model protein by causing backtracking events resulting of an untimely acquisition of the native conformation. In order to investigate further the impact of a structured terminus in the folding of knotted proteins we evaluated the folding rates of the control systems (Figure S2). With the exception of the k1-c1 pair, for which the folding rates are similar, our results indicate that the establishment of native contacts by the terminal beads systematically leads to slower folding rates, most likely resulting from topological traps forming prior to the threading of the knotted core by the chain terminus. They also highlight the importance of the structured terminus in the k3h knot which folds 1.3 times slower than the control system c3 (with k3l folding 1.2 times slower than c3). In summary, increasing the knot's depth by extending the size of the protein terminus has a major effect in decreasing the folding rate. However, the native interactions involving a structured chain terminus also contribute to slow down the folding process presumably by causing backtracking events.

### Knot deepness and unfolding rate

The unfolding rate provides a measure of kinetic stability, a property of the native state that is essential to maintain the biological function of the protein, at least during a physiologically relevant time-scale [50]. An enhanced kinetic stability may be particularly relevant for proteins forming transmembrane channels that are subjected to mechanical stress. Since knotted patterns appear to be preferentially conserved among transmembrane channels [5] one may speculate that the functional role of knots in proteins is to enhance their kinetic stability. Motivated by this idea, we investigated the relation between knot deepness and kinetic stability. In order to do so, we measured the unfolding rate of the knotted (Figure 3C) and unknotted (Figure 3D) systems in temperature conditions that thermodynamically stabilize the denatured state relative to the native state, i.e., at 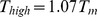. Our results reveal that if the knot is shallow (as in k0), the ratio between the unfolding rate of the unknotted protein and that of its knotted homologue, 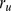, is close to unity (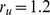) indicating that a protein with a shallow knot unfolds at a rate similar to that of the homologous unknotted protein. As knot deepness increases 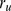 also increases (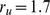 for the k1/u1 pair, 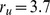 for the k2/u2 pair and 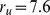 for the k3l/u3l pair), indicating an increase in kinetic stability. However, the largest enhancement in kinetic stability was not recorded for the deepest knot studied here kd (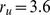) but for the knot k3h (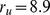) instead. An important difference between knots k1, k2, k3l, k3h and knot kd is the fact that in the native structures of the first four knots the chain termini are docked onto each other. In k3h the terminus is furthermore arranged into a hairpin like conformation. In kd, on the other hand, the termini are fairly separated and arranged in a linear manner. Thus our results suggest that the conformation adopted by the termini in the native structure is an important determinant of kinetic stability, being more important than knot depth at high temperature.

We further investigated the kinetic stability of k0, k1, k3h and kd in temperature conditions that thermodynamically stabilize the native state, i.e., at 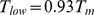. The results we obtain for k0 (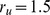) and k1 (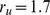) are similar to those obtained at a higher temperature, while the enhancement in kinetic stability for k3h (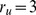) is not as striking than that recorded at 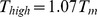. On the other hand it becomes remarkably more difficult to unfold the deeper knot kd at a lower temperature (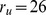), suggesting that knot deepness may become more important as a driver of kinetic stability below 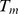. Further studies, exploring an enlarged set of diverse knotted conformations, should be carried out in order to ascertain the importance of these structural aspects - knot deepness and structured termini - as determinants of kinetic stability in knotted proteins.

We evaluated the probability of knot formation in the unfolded state of all the knotted systems at 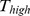. We observe that 2% (k0), 11% (k1), 38% (k2), 79% (k3l), 78% (k3h) and 98% (kd) of the denatured conformations in the denatured ensemble were knotted. At 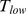 we get 1% (k0), 3.6% (k1), 45% (k3h) and 98% (kd). Both trends indicate that the probability to keep knotted in the denatured state increases with knot deepness. The denatured ensemble of the deep knot kd displays the same high knotting probability. On the other hand, the probability to find a knotted backbone in the denatured ensembles k1 and k3h decreases. The observation that a higher temperature does not necessarily increase the probability of unknotted denatured conformations state indicates that it is unlikely to unknot a protein by means of a random process.

### The denatured ensemble of knotted proteins

**Figure 4:**
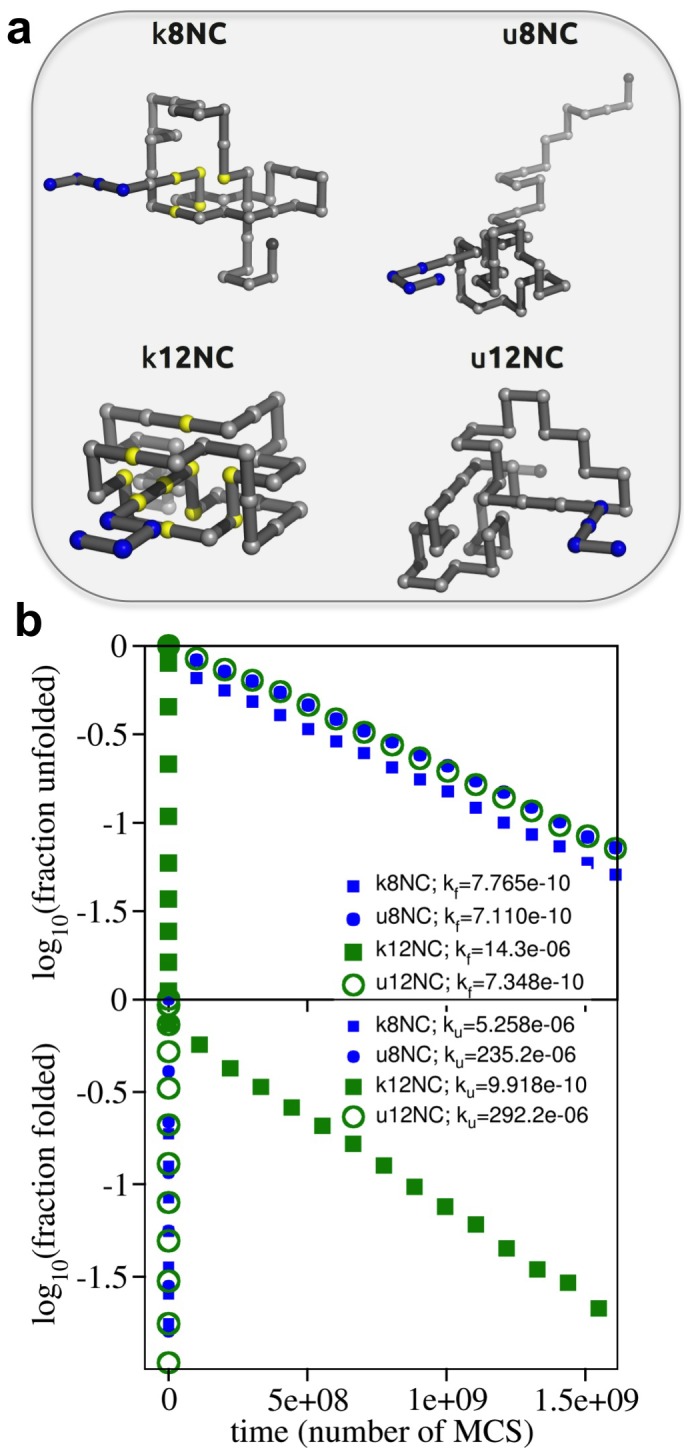
Impact of knot nativeness on folding and unfolding efficiency. Panel (a) shows two knotted conformations with 8 (k8NC) and 12 (k12NC) native contacts that were sampled from unfolding simulations. Beads colored in yellow have at least two of its native contacts established. Three native contacts in k8NC pertain to the knotted core; k8NC also has an incorrect crossing and this malformed conformation classifies as a topological trap. On the other hand, the k12NC conformation has 7 native contacts established that belong to the knotted core and part of the knotting loop is already in its native position. This conformation classifies as a native knot. The two unknotted conformations shown in the figure, u8NC and u12NC, were also sampled from unfolding simulations and have 8 and 12 native contacts formed. Panel (b) reports the measurement of the folding (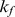) and unfolding (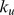) rates starting from these conformations. The unfolding rate of k12nc is six orders of magnitude smaller than those of the unknotted ones and four orders of magnitude smaller than that of k8nc. In the protein backbones is knotted as in 8knc, the unfolding rate becomes two orders of magnitude smaller with respect to the unknotted conformations.

The occurrence of knotted conformations in chemically denatured states was recently reported [15]. It has been suggested that the in vitro folding efficiency of chemically denatured knotted proteins (which exhibit refolding rates of the same order of magnitude of proteins without knots) could, in fact, be a direct consequence of persisting knotted conformations in the denatured state. While these conformations contain the native knot, the properties of the knot remain to be elucidated (e.g. whether the knot is tight or loose, stabilized by native or non-native interactions etc.). Therefore it is important to establish if folding performance depends on the knot's properties. In what follows we investigate the extent up to which the folding rate depends on the number of established native contacts pertaining to the knotted core. In order to do so we started by performing structural clustering on two ensembles of conformations: one in which the conformations have *Q* = 0.18, and a second one in which the conformations have *Q* = 0.27 and extracted the representative conformations (Figure 4A). The representative conformation of the first ensemble is termed k8nc. Although three of its native contacts belong to the knotted core (which contains eight native contacts (Figure S1)), this conformation exhibits an incorrect crossing, and therefore is classifies a being a topological trap [23]. The representative conformation of the second ensemble is termed k12nc. It has no incorrect crossing and five of its 12 native contacts belong to the knotted core. With the purpose of carrying out control experiments, we have also selected two unknotted conformations, one with eight native contacts and another with 12 native contacts (Figure 4A) that belong to the clusters form which we extracted the knotted ones. The folding rate of the four conformations was evaluated. The results are reported in Figure 4B show that the folding rate of the conformation in which the knotted core is more consolidated is about five orders of magnitude larger than that of the other conformations (which display similar folding rates) and 1.2 orders of magnitude larger than the folding rate of k3h starting from an unfolded conformation. This indicates that if the knotted core's native interactions persist in denatured conformations, refolding proceeds very fast. On the other hand, if the denatured conformation contains an incorrect crossing such as that occurring in k8nc, then the folding rate is exceedingly slow. Indeed, a detailed analysis of the folding kinetics curve reveals the occurrence of non-linear behavior at early *t*, which may be taken as an indication of large backtracking events which are required to correct the wrong crossing prior to entering the correct folding pathway (Figure S3). This result is in agreement with the idea that folding nucleation by non-specific knots is entropically unlikely [23].

In order to investigate if it is easier to unknot the protein backbone starting from a denatured-like conformation we ran unfolding simulations starting from the four analyzed conformations. We observe that the unknotted conformations unfold faster than the knotted ones and with similar rates. On the other hand, a more consolidated knotted core, as in k12nc, dramatically slows down unfolding. The probability to find a knotted conformation in the denatured ensembles of k8nc and k12nc is similar and moderately high (∼0.7), which shows that it is remarkably difficult to unknot the protein backbone, even if the temperature is higher than the physiological temperature. We thus conclude that the denatured ensemble is highly resilient in what concerns the conservation of its knotted topology.

### Identification of native contacts that nucleate the knot

The study of conformation k12nc shows that if the native interactions within the knotted core are preserved in denatured conformations folding to the native state proceeds fast and efficiently. But are all the native interactions in the knotted core equally important to nucleate folding? In order to address this question we investigated the denatured ensemble of kd (obtained at 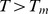) to search for contacts forming with high probability in knotted conformations. Such contacts may be putative nucleation spots for the knot. The probability map (Figure S4) reveals the presence of a contact (between beads 11 and 28) that forms with probability ∼0.5 and belongs to the knotted core. Other relevant contacts belonging to the knotted core (11–26 and 13–28) are present with probability ∼0.3–0.4. In order to investigate the importance of persistent native contacts in folding kinetics we have randomly selected - from the ensemble of 2000 conformations used to construct the probability map - the first conformation with more than one knotted core contact formed (we recall that each one of these denatured conformations only has four native contacts). The knotted core contacts turned out to be 11–28 and 11–26. Interestingly, in the selected conformation, the formation of contact 13–28 is only one corner-flip move away. An important trait of this conformation is the preservation of the knotting loop (Figure S5). Quite remarkably we found that 87% of the 2000 MC runs found the native state in less than 1×10^7^ MCS with a folding rate (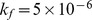) which is four orders of magnitude larger than the folding rate starting from unfolded conformations (Figure S5). This result shows that denatured conformations with a very small amount of native contacts established will fold dramatically fast if they preserve important structural features such as the knotting loop. It also highlights the importance of specific native contacts in structurally stabilizing the knotting loop. Thus, if the denaturing experiments do not break these important native interactions refolding will proceed very fast. This finding suggests that specific contacts involving the threading terminus and the knotting loop will have an important role in folding by nucleation of knotted proteins.

### Knot deepness and knotting probability

**Figure 5:**
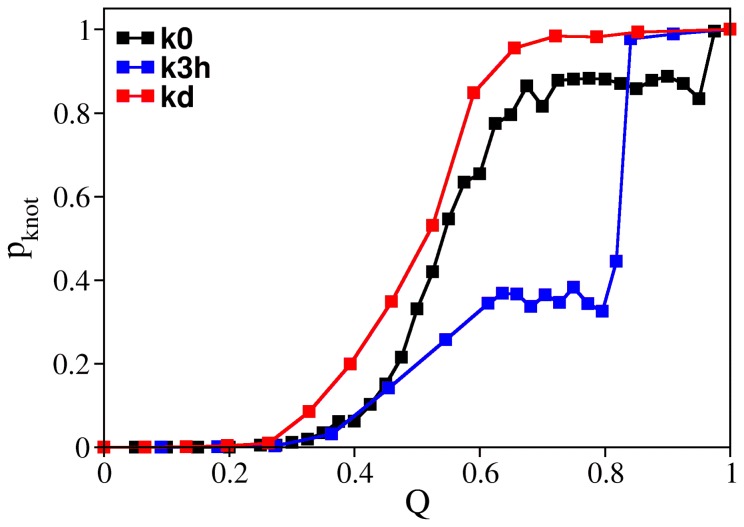
Probability of knot formation as a function of the reaction coordinate, *Q*, the fraction of established native contacts for systems k3h and kd. Also shown is the curve for the reference system k0.

We now turn our attention to the knotting mechanisms of model proteins k3h and kd. For that we start by measuring the knotting probability 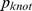 as a function of the reaction coordinate fraction of native contacts, *Q*. To carry out this measurement we have constructed an ensemble of 2000 (uncorrelated) conformations for each fraction of native contacts *Q* that were collected from many independent MC runs. The KMT algorithm was applied to each conformation to investigate the presence of the knot (Figure 5). In the case of k3h, there is a plateau between *Q* = 0.6–0.8 in which 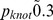. As we will show, k3h gets trapped into a series of misfolded conformations during its folding process that are responsible for the occurrence of the plateau in the 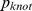 curve. On the other hand, we obtain a sigmoidal curve for kd, which is qualitatively similar to that of the shallow knotted protein k0. Interestingly, the knotting probability in kd is systematically larger than in the other knotted systems, which indicates that an increase in size of the threading terminus enhances knotting efficiency.

### Knot deepness and knotting mechanism

In order to gain further insight into the structural changes occurring during folding we performed hierarchical clustering (based on contact-map similarity) over ensembles of conformations with increasing *Q*, and we have extracted the representative conformation (i.e. the one closest to the average contact map) from each cluster's centroid. As before, each starting ensemble contains 2000 uncorrelated conformations that were collected from many independent folding runs. We have thus obtained a succession of conformations of increasing *Q* that provides insight into the structural changes underlying the knotting mechanism at play for model systems k3h and kd.

### Knot k3h

**Figure 6:**
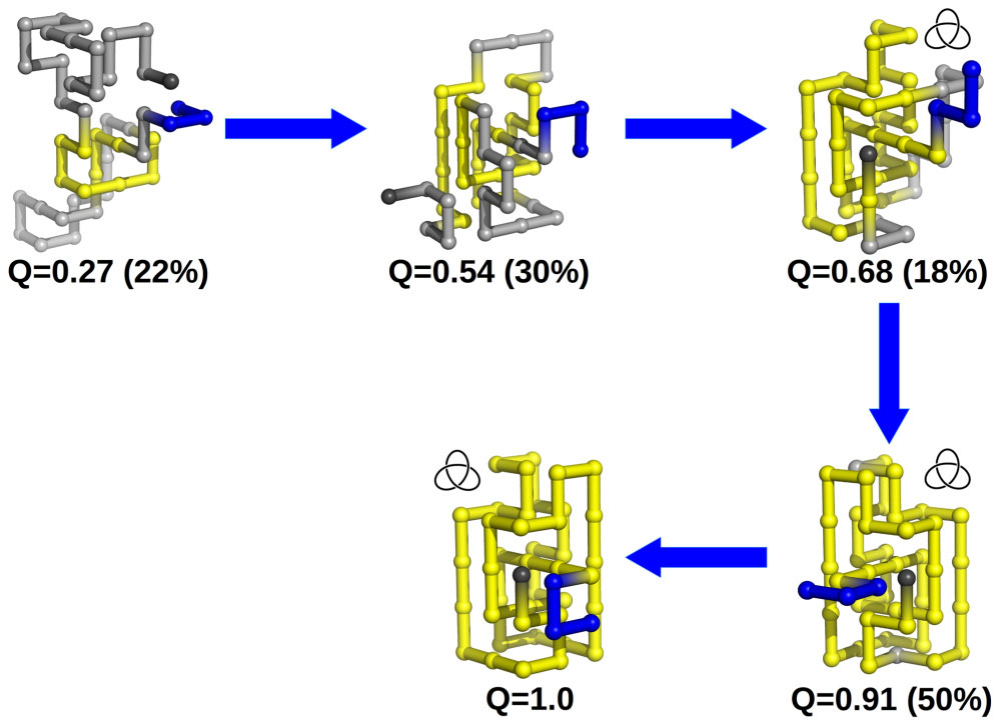
Insights into the knotting mechanism of k3h from structural clustering. Each conformation with fraction of native contacts *Q* is the closest to the cluster's centroid, and is taken as the cluster's representative. The residues colored in yellow have at least two of its native contacts formed. In parenthesis we show the ratio between the size of the cluster (i.e. its number of conformations) and the size of the initial ensemble of conformations with fraction of native contacts *Q*. In this mechanism knotting occurs in a conformation with fraction of native contacts 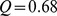. The chain terminus closest to the knotted core threads a knotting loop that is already in its native conformation. The hairpin-like terminus only acquires its native conformation after threading.

We have identified two folding pathways for the k3h knot, which differ in the stage at which the knotting of the protein backbone takes place during folding. In the late threading pathway the chain terminus threads a knotting loop that is already in the native conformation (Figure 6). In the early threading pathway the backbone gets knotted in conformations with smaller *Q*, i.e., when the protein has a smaller fraction of native contacts established (Figure S6). The size of the identified clusters indicates that the late threading mechanism is more likely than the early threading one. This is not surprising because an early threading pathway will have a free energy barrier with a large entropic contribution resulting from the little energetic stabilization until the native environment forms around the knot. This is particularly true in the case of the Gō potential that ignores non-native interactions. The latter could have a stabilizing role by balancing the entropy loss occurring in a knotting mechanism based on an early threading event.

The two identified pathways are similar to those we have previously identified for the shallow knot k0 [19]. However, the increased size of the terminus in k3h has an important consequence regarding the folding mechanism of this knotted protein: it leads to the formation of many misfolded conformations with fraction of native contacts *Q* between 0.6 and 0.8 (Figure S7, Figure S8 and Figure S9). The formation of these topological traps results into the knotting probability plateau within this *Q* region (Figure 5). These conformations are energetically close to the native state but they are topologically distant from the native conformation, which can only be achieved if a specific (and highly unlikely) chain crossing occurs [23] (Figure S7). It is interesting to note that we have also found evidence for a misfolding pathway that is not associated to incorrect threading. Indeed, the misfolded conformations form prior to threading and actually impede it because they result from the precocious establishment of native interactions involving the chain terminus (Figure S8 and Figure S9). This observation justifies the slowing down of the folding process of knot k3h relative to the control system c3 (Figure S2).

### Knot kd

**Figure 7:**
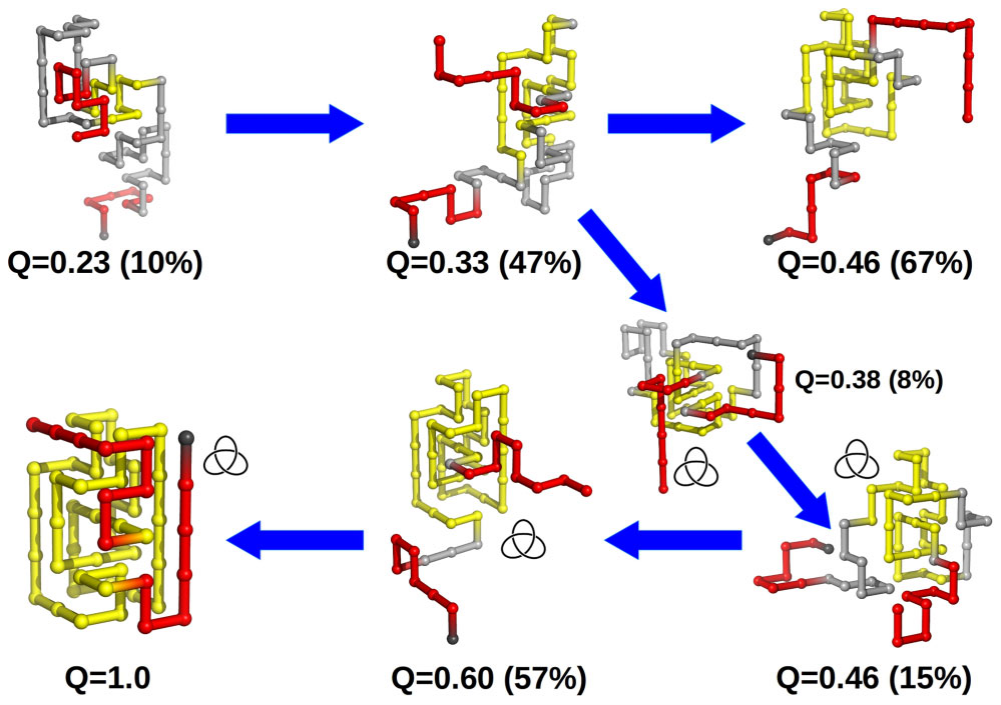
Insights into the knotting mechanism of kd from structural clustering. There is an important conformational state with fraction of native contacts 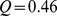 that corresponds to a malformed conformation with an incorrect crossing of the threading terminus, which nevertheless has a rather native-like knotting loop. Productive threading requires an enlarged and loosen knotting loop and occurs in conformations with fraction of native contacts 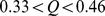.

The folding pathways of the kd system were investigated in a similar manner. In this case we also found evidence for an early-threading pathway, in which the chain terminus threads a knotting loop which is partially stabilized by native interactions, but the threading terminus itself does not establish any native interactions with the reminder of the chain (Figure S10). The relatively small size of the identified clusters suggests that this pathway occurs with low probability; the reason is the same as that put forward in the case of k3h. On the other hand, the dominant pathway appears to be based on a threading event occurring in conformations that are more nativelike (Figure 7). While, the identification of these conformations was not straightforward our results suggest that they have a fraction of native contacts *Q* between 0.3–0.4 and, more importantly, their knotting loop is substantially larger (i.e. loosen) than that of k0 and k3h. This structural requisite results from the larger size of the threading terminus in the case of kd. It is quite likely that this structural trait is precisely what makes the identification of the knotting state via clustering more challenging. Indeed, it is possible that prior to threading the chain needs to visit a large number of conformations until it finds one with an appropriate knotting loop. This increase in structural fluctuations and the diversity of conformations it generates prior to threading makes the isolation of specific conformational states more difficult. In line with this hypothesis we find the occurrence of topological traps for *Q* = 0.46 resulting from an incorrect threading of the terminus leading to incorrect crossings (Figure S11 and Figure S12). However, we cannot exclude the possibility that, at least in part, trapped conformations are due to the strong geometric restrictions imposed by the lattice, and lack of an explicit chirality term in the adopted interaction potential [51].

## Conclusions

This work explored the impact of knot depth in protein folding properties and mechanism via extensive Monte Carlo simulations of lattice models. In particular, starting from a shallow trefoil knot, it presents a systematic investigation of the impact of knot depth on folding performance by using an array of computational tools. It also investigates the role of chain termini in the folding of knotted proteins by considering two knotted structures: one in which the threading terminus is a linear segment and another one in which the threading terminus is folded into a hairpin like conformation but the knot is not as deep. We found that the existence of a knot does not lead to a noticeable increase in thermal stability. On the other hand, the folding rate is severely hampered by the knot's presence. Interestingly, a structured terminus can have a more stringent effect on the decrease of folding rate than knot deepness. Our results indicate that the most important (and presumably most advantageous) effect of knots in protein properties is an increase in kinetic stability. They also show that if the temperature is higher that the folding temperature a structured threading terminus can be more effective than knot depth in enhancing the kinetic stability of knotted proteins.

A rather important observation we draw from this work regards the difficulty in untying the knotted backbone once it is formed. Indeed, we have found that the most probable topology of the denatured state of knotted proteins is a knotted state, and knot depth tends to increase the likelihood of denatured knotted conformations. This is in line with experimental data showing that knots persist in denatured conformation forming in highly denaturing conditions [15], and also with previous simulation results by Noel et al. [23]. All together these results points out to an hypothesis of Mallam and Jackson according to which threading only occurs once after protein synthesis and in subsequent unfolding and refolding events the protein chain remains knotted in state that may reflect kinetic trapping [16]. These observations indicate that knotted proteins cannot be randomly untied. Microscopically speaking the existence of a knot in the protein backbone imposes steric constraints that severely restrict conformational sampling. Breaking interactions in the knotted core does not by itself remove these constraints, which will persist for conformations where the topological loop is still present and the knot is still tight. For these conformations, en route to unknotting, the entropy gain that could compensate enthalpic destabilization is limited, making the unknotting process, overall, not thermodynamically favoured.

The results presented here regarding refolding processes starting from knotted conformations show that not every denatured knotted conformation is able to nucleate folding. Instead, knot specificity is essential to drive an efficient and fast folding process, and knots with at least one incorrect crossing (topological traps) strongly hamper folding efficiency because they require large backtracking events. However, and more interestingly, our results also show that knot specificity is compatible with a denatured state with no detectable structure. Indeed, a conformation that keeps the knotting loop but has a very small fraction of established native contacts (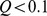) is able to nucleate folding with a folding speed that is dramatically larger than that recorded in folding experiments starting from unknotted conformations, highlighting the importance of native loop formation in the folding of knotted trefoils. The preservation of such conformations in the denatured ensemble appears to be the result of resilient native contacts, which belong to the knotted core, and may act as nucleation spots in the folding of knotted proteins.

The study of the folding and knotting mechanism points out to the most likely scenario being the formation of the knot towards late folding in preordered conformations where the knotting loop is substantially consolidated or already in its native conformation. The knot forms when the terminus that is closer to the knotted core threads the knotting loop. Interestingly, although in one of the studied model proteins the threading terminus adopts a hairpin-like conformation in the native state, we have not observed a knotting mechanism based on the formation of a slipknot. Instead, the existence of a structured terminus has an important effect in creating malformed conformations that are characterized by an untimely docking of the structured terminus onto the other terminus prior to threading. Although misfolded states are also observed in the folding of the protein with the deepest knot investigated here, in both cases they are transiently populated as they do not show up in the thermodynamic data, namely, in the heat capacity curves and free energy profiles (data not shown).

## Supporting Information

Figure S1 Native contact maps and native structures. The region of the native structures colored in grey represent the original native structures k0 and u0 from which we constructed kd (a) and ud (b) by extending both termini, and the other model systems by extending the termini closest to the knotted core (colored green). The extended termini are colored in red and the contacts they establish are colored in red in the contact maps. The knotted core (colored green) comprises residues 11 to 30 and 8 native contacts (11–26, 11–28, 13–28, 13–30, 15–30, 16–29,18–29 and 20–23). These are marked with circles in the contact map of the knotted structure (a).
(TIF)

Figure S2 Comparison of the folding rates exhibited by the knotted systems k and those of the control systems. The control systems also have their backbone knotted but the chain terminus closest to the knotted core is not involved in native interactions. Also shown is the folding rate of the original system that contains a shallow knot, k0.
(TIF)

Figure S3 Comparison between the folding kinetics of knotted conformation k8NC with unknotted conformations u8NC and u12NC. The non-linear behavior at early time in the curve suggests the occurrence of a long-lived state for k8NC from which is difficult to escape due to an incorrect crossing.
(TIF)

Figure S4 Probability map showing the frequency of occurrence of intramolecular contacts in the denatured state of model system kd. To compute this map we constructed an ensemble of 2000 conformations, where each conformation has four or less native contacts. These conformations were selected from many independent MC runs being therefore uncorrelated.
(TIF)

Figure S5Refolding from a knotted denatured conformation with 4 native contacts. The conformation shown in (Figure A) was obtained from unfolding simulations at 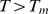, displays and intact knotting loop which is structurally stabilized by two key contacts (11–28 and 11–26). The vast majority of folding simulations starting from this conformation are dramatically fast reaching the native state in less than 10^7^ MC-steps (Figure B).(TIF)

Figure S6 Alternative folding pathway for kh3. Here and in the following figures each displayed conformation with fraction of native contacts *Q* is the closest to the cluster's centroid, i.e., the cluster's representative. The residues colored in yellow have at least two of its native contacts formed. In parenthesis we report the ratio between the size of the cluster (its number of conformations) and the initial ensemble from which they were clustered where conformations have fraction of native contacts *Q*. In this folding pathway the knotting of the protein backbone occurs in conformations with *Q* = 0.45. On the other hand, in the dominant (i.e. most likely) folding pathway (Figure 8) the threading of the knotting loop occurs in conformations with a larger fraction of native contacts (*Q* = 0.68).
(TIFF)

Figure S7 Formation of misfolded conformations in kh3 due to incorrect threading of the terminus.
(TIFF)

Figure S8 Pathway in kh3 leading to dead-end conformations.
(TIFF)

Figure S9 Pathway in kh3 leading to misfolded conformations. In these misfolded conformations the threading terminus docks onto the other terminus forming native contacts that stabilize the conformation.
(TIFF)

Figure S10 Alternative folding pathway for kd. In this folding pathway the knotting of the protein backbone occurs in conformations with *Q* = 0.33.
(TIFF)

Figure S11 Formation of misfolded conformations in kd due to incorrect threading of the terminus.
(TIFF)

Figure S12 Formation of misfolded conformations in kd due to incorrect threading of the terminus.
(TIFF)
